# Specific protonation of acidic residues confers K^+^ selectivity to the gastric proton pump

**DOI:** 10.1016/j.jbc.2023.105542

**Published:** 2023-12-10

**Authors:** Hridya Valia Madapally, Kazuhiro Abe, Vikas Dubey, Himanshu Khandelia

**Affiliations:** 1 PHYLIFE, Physical Life Science, Department of Physics Chemistry and Pharmacy, University of Southern Denmark, Odense, Denmark; 2 Graduate School of Pharmaceutical Sciences, Nagoya University, Nagoya, Japan; 3 Cellular and Structural Physiology Institute, Nagoya University, Nagoya, Japan

**Keywords:** proton pump, H^+^,K^+^-ATPase, membrane protein, ion selectivity, cryo-EM, molecular dynamics, free energy perturbation

## Abstract

The gastric proton pump (H^+^,K^+^-ATPase) transports a proton into the stomach lumen for every K^+^ ion exchanged in the opposite direction. In the lumen-facing state of the pump (E2), the pump selectively binds K^+^ despite the presence of a 10-fold higher concentration of Na^+^. The molecular basis for the ion selectivity of the pump is unknown. Using molecular dynamics simulations, free energy calculations, and Na^+^ and K^+^-dependent ATPase activity assays, we demonstrate that the K^+^ selectivity of the pump depends upon the simultaneous protonation of the acidic residues E343 and E795 in the ion-binding site. We also show that when E936 is protonated, the pump becomes Na^+^ sensitive. The protonation-mimetic mutant E936Q exhibits weak Na^+^-activated ATPase activity. A 2.5-Å resolution cryo-EM structure of the E936Q mutant in the K^+^-occluded E2-Pi form shows, however, no significant structural difference compared with wildtype except less-than-ideal coordination of K^+^ in the mutant. The selectivity toward a specific ion correlates with a more rigid and less fluctuating ion-binding site. Despite being exposed to a pH of 1, the fundamental principle driving the K^+^ ion selectivity of H^+^,K^+^-ATPase is similar to that of Na^+^,K^+^-ATPase: the ionization states of the acidic residues in the ion-binding sites determine ion selectivity. Unlike the Na^+^,K^+^-ATPase, however, protonation of an ion-binding glutamate residue (E936) confers Na^+^ sensitivity.

Stomach acidity is maintained by the P-type ATPase protein called H^+^,K^+^-ATPase (1). It transports protons from the stomach parietal cells to the gastric lumen against a million-fold concentration gradient and requires energy from ATP to function (2). The process is kept electroneutral by transporting extracellular K^+^ ions into the parietal cells from the gastric lumen (3). H^+^,K^+^-ATPase contains two subunits—the α-subunit, which transports the ions across the membrane, and the β-subunit, which helps in membrane targeting and regulation of protein conformational equilibrium (4, 5). The α-subunit contains ten transmembrane helices that constitute the ion-binding site and three cytoplasmic domains. The N domain binds to ATP, the P domain contains the amino acid (Asp385) that is phosphorylated by ATP, and the A domain facilitates dephosphorylation of phosphorylated Asp385. The transport occurs *via* a cyclical conformational change of the protein conforming to the Post–Albers cycle (6, 7), which was first described for Na^+^,K^+^-ATPase, another member of the P-type ATPase family. The transport ensues through two major conformations of the protein: E1 and E2. In the E1 state, the ion-binding site is open to the cytoplasm and has a higher affinity to bind a proton than K^+^. In the E2 state, the ion-binding site faces the gastric lumen and has a higher affinity to bind a K^+^ than a proton, resulting in release of the proton to the lumen. The binding of a proton to the protein in the E1 state induces the phosphorylation of the Asp385 in the P domain. This initiates a protein conformational change resulting in the formation of the E2-P configuration, leading to the release of proton and consequent binding of K^+^. The binding of K^+^ triggers the dephosphorylation of the phosphorylated Asp385, which subsequently prompts conformational changes in the protein to form the E1 state and the successive release of the K^+^ ion into the cytoplasm.

Although Na^+^ ions (70–120 mmol/L) are present at much higher concentration than K^+^ ions (10 mmol/L) in the gastric lumen (8), H^+^,K^+^-ATPase is selective toward the binding of K^+^. The mechanism by which H^+^,K^+^-ATPase achieves a high degree of selectivity in binding to K^+^ ion in the E2 state remains unknown. The Na^+^,K^+^-ATPase, whose α-subunit is ∼65% homologous to that of the H^+^,K^+^-ATPase (9), transports Na^+^ and K^+^ ions across the membrane against their concentration gradient under physiological conditions. It binds Na^+^ in the presence of 10-fold higher concentration of K^+^ ions and binds K^+^ in the presence of 30-fold higher concentration of Na^+^ ions. The ionization states of acidic residues at the ion-binding site of Na^+^,K^+^-ATPase play an important role in selectively recognizing the ion at each stage of the cycle (10, 11, 12, 13). A decrease in pH leads to an increase in the K^+^ binding affinity, which possibly arises from the change in ionization state of the coordinating residues (10). Molecular dynamics simulations showed that protonation of acidic residues at the cation-binding site is critical for ion selectivity (11, 12). A recent investigation indicates that the changes in the protonation states of acidic residues arises from the conformation-dependent binding of an anion to the protein (13). While K^+^ selectivity is influenced by the protonation state, Na^+^ selectivity is governed by the steric constraints in the binding site (13). Since Na^+^,K^+^-ATPase and H^+^,K^+^-ATPase are highly homologous, it is probable that the ionization state of the key acidic amino acids at the ion-binding site plays a crucial role in the K^+^ selectivity of the H^+^,K^+^-ATPase too. However, this hypothesis has not been previously validated. Moreover, the extracellular domain of H^+^,K^+^-ATPase is exposed to a pH of 1, as opposed to 7 in the case of the Na^+^,K^+^-ATPase. Given that the pH will influence the ionization state of the binding site anionic residues, it is possible that the mechanism that confers K^+^ selectivity to Na^+^,K^+^-ATPase is different from that of H^+^,K^+^-ATPase.

The two K^+^ ion-binding sites in the Na^+^,K^+^-ATPase are designated site I and site II. K^+^ occupies the corresponding site II in H^+^,K^+^-ATPase in the crystal structure of K^+^-bound H^+^,K^+^-ATPase (14). A salt bridge between Lys791 and Glu820 spatially limits the binding of K^+^ to site I. There are eight oxygen atoms located within 4 Å of the bound ion. Val338, Ala339, and Val341 coordinate through their main-chain carbonyl oxygen atoms, while Glu343 and Glu795 coordinate *via* their side-chain carbonyl oxygens. Four other acidic amino acids, Glu820, Asp824, Glu936, and Asp942, also lie in close proximity to the binding site. Given the close proximity of the six acidic residues, it is reasonable to assume that some of them must remain protonated to stabilize the binding site. Using structural data, molecular dynamics simulations, and biochemical analysis of mutations in the ion-binding site, we previously hypothesized that Glu343, Glu795, and Glu936 are likely to remain protonated during K^+^ binding (14).

Here, we systematically investigate the effect of ionization states of the acidic amino acids at the ion-binding site on the selective binding of K^+^/Na^+^ in the E2P conformation. We employ the free energy perturbation method to calculate relative binding free energies of Na^+^ and K^+^ for various combinations of ionization states of acidic residues in the ion-binding site. We refer to these combinations as “protonation states” from here on. Our simulations are complemented by K^+^ and Na^+^-dependent ATPase activity measurements of protonation-mimicking mutants of the H^+^,K^+^-ATPase. We analyzed the cryo-EM structure of protonation-mimetic E936Q mutant and found no significant differences from previously reported wildtype structure. We find that the protonation of the residues E343 and E795 is a prerequisite for K^+^ selectivity. Furthermore, the protonation of E936 tends to make the pump less K^+^ selective.

## Results and discussion

### Relative binding affinity from free energy perturbation calculations

There are six acidic residues that are in close proximity to the ion-binding site (14). The residue E343 is in TM4, E795 is in TM5, E820 and D824 are in TM6, and E936 and D942 are in TM8. Of these, E343, E795, and E820 directly coordinate the ion at the binding site while D942 is the furthest from the ion-binding site. We first estimate the ionization states of these residues in the K^+^ bound crystal structure of the protein using the PROPKA (15) algorithm (Table 1). The unusually high pKa of E795 can be attributed to the close proximity of the acidic amino acid E820 to E795. When both amino acids are deprotonated (have low pKa), there is coulombic instability in the binding site. Therefore, one residue is protonated and must have a high pKa. Furthermore, E820 remains deprotonated and forms a salt bridge with K791. Furthermore, PROPKA predicts that E343, E795, E936, and D942 remain protonated at a pH of 7, in agreement with the protonation state predicted by Yamamoto *et al.* (14) for the E2 state of the protein. To ascertain whether this specific protonation state indeed governs the selective binding of K^+^, we calculated the difference in free energy of binding of various combinations of ionization states for these residues.

**Table 1:** pKa values of the titratable residues as predicted by the PROPKA algorithm from the K^+^ bound crystal structure

Residue	pKa
E343	10.62
E795	13.15
E820	4.60
D824	5.36
E936	10.73
D942	9.07

EGFP, enhanced green fluorescence protein; FEP, free energy perturbation; TEV, tobacco etch virus.

**Figure 1:**
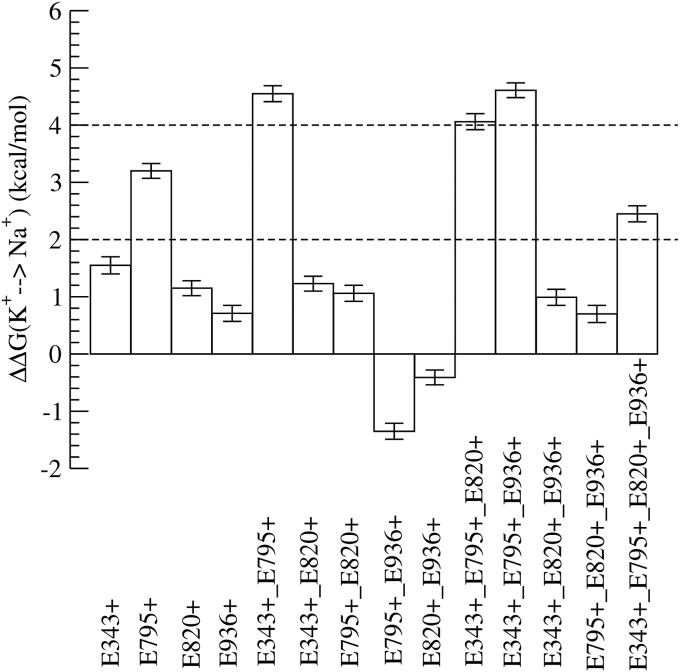
**The**${\Delta \Delta G}_{binding}^{\left(K^{+}\to {Na}^{+}\right)}$**for the different protonation states obtained from the free energy perturbation calculations**.

An exhaustive calculation of the difference in binding energy of K^+^ and Na^+^ for all 64 combinations of protonation states of the amino acids is superfluous. We disregard some protonation states based on their relevance to binding site coordination. We do not consider different ionization states of D942 because it lies furthest from the ion-binding site and is unlikely to affect the ion coordination. Secondly, the residues E820 and E795 are in close proximity and we assume that at least one of them is always protonated. Finally, we neglect the ionization states of D824 because the D824N mutant had negligible ATPase activity, as explained later. For the remaining protonation states, the difference in free energy of binding for Na^+^ and K^+^ determined from the free energy perturbation (FEP) calculations is shown in Figure 1. Convergence of the FEP simulations was ensured from the difference in the free energy between the forward and reverse free energy calculations (Fig. S1). Note that the FEP free energy perturbation does not provide absolute free energy binding values but the relative difference in free energy of binding of the two ions, ${\Delta \Delta G}_{binding}^{\left(K^{+}\to {Na}^{+}\right)}$. We would like to remind the reader that a positive value of ${\Delta \Delta G}_{binding}^{\left(K^{+}\to {Na}^{+}\right)}$ indicates higher affinity to bind K^+^ than Na^+^, whereas a negative value of ${\Delta \Delta G}_{binding}^{\left(K^{+}\to {Na}^{+}\right)}$ indicates a higher affinity to bind Na^+^ than K^+^. Based on the values of ${\Delta \Delta G}_{binding}^{\left(K^{+}\to {Na}^{+}\right)}$, we classify the different protonation states of the protein into three categories: K^+^ selective, mildly K^+^ selective, and Na^+^ sensitive:

(1) A state is classified as K^+^ selective when ${\Delta \Delta G}_{binding}^{\left(K^{+}\to {Na}^{+}\right)}>4\;kcal/mol$, because the likelihood to bind K^+^ is higher than 600 times compared with that of Na^+^.
(2) When ${\Delta \Delta G}_{binding}^{\left(K^{+}\to {Na}^{+}\right)}<2\;kcal/mol$, then the state is classified as Na^+^ sensitive. The pump can bind either ion because the free energy difference is comparable with thermal energy: *k*_*b*_*T*, and the error in the calculations.
(3) Finally, when $2\;kcal/mol\;<\;{\Delta \Delta G}_{binding}^{\left(K^{+}\to {Na}^{+}\right)}<4\;kcal/mol$, we classify the pump as mildly K^+^ selective.

### K^+^-selective protonation states ${(\Delta \Delta G}_{binding}^{\left(K^{+}\to {Na}^{+}\right)}>4kcal/mol)$

**Figure 2:**
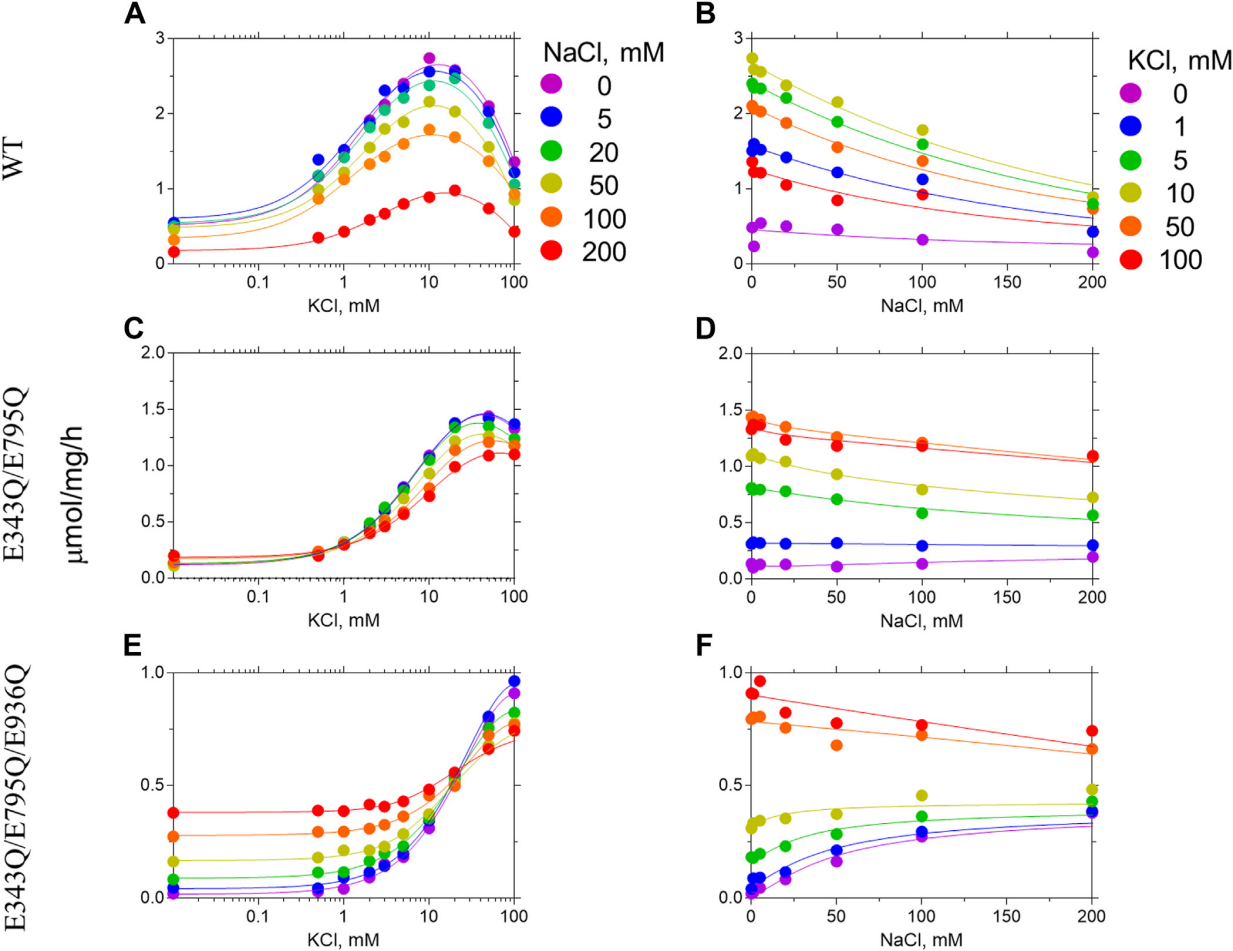
**ATPase activity assays.** K^+^-dependent (*A*, *C*, and *E*) and Na^+^-dependent (*B*, *D*, and *F*) ATPase activity in crude membrane fractions from cells expressing wildtype (WT, *A* and *B*), E343Q/E795Q double mutant (*C* and *D*), and E343Q/E795Q/E936Q triple mutant (*E* and *F*) in the presence of indicated concentrations of Na^+^ or K^+^ (color coded in the figure). The line plots are fits of a double Hill equation to the data. See Experimental procedures for details.

E343 and E795 are protonated in all the distinctly K^+^-selective protonation states. These are the protonation states E343^+^_E795^+^, E343^+^_E795^+^_E820^+^, and E343^+^_E795^+^_E936^+^. Before comparing our results with ATPase activity assays, we would like to describe the assay to the reader. The Na^+^ and K^+^-dependent ATPase activity of the wildtype protein is shown in Figure 2, *A* and *B*. The wildtype pump has almost very low activity at [KCl] = 0 mM (Fig. 2*A*), which can be attributed to background ATPase activity due to spontaneous dephosphorylation of the E2P state independent of K^+^ binding. However, this activity is suppressed by Na^+^ ions (see Fig. 2*B* purple circles) as the binding of Na^+^ cannot induce phosphorylation. The pump activity initially increases with increase in [K^+^] and decreases due to reverse inhibition beyond [K^+^] = 20 mM. Decrease in activity with increasing [Na^+^] (Fig. 2*A*) shows that Na^+^ can compete for binding at higher [Na^+^] but cannot activate the pump. For all nonzero [K^+^], pump activity is inhibited by increasing [Na^+^], confirming that Na^+^ binds to the pump without activating it. The experimentally estimated ${\Delta \Delta G}_{binding}^{\left(K^{+}\to {Na}^{+}\right)}$ for the wildtype is between 2.69and3.12kcal/mol, which compares favorably with the numbers calculated from the simulations. E343Q_E795Q (corresponding to E343^+^_E795^+^ in the simulations) has behavior similar to that of WT (Fig. 2, *C* and *D*), supporting the simulation inference that E343 and E795 must be protonated for K^+^ selectivity. However, in E343Q_E795Q_E936Q (corresponding to E343^+^_E795^+^_E936^+^ in simulation), the pump activity increases with [Na^+^] at low [K^+^] regimens (Fig. 2*F*), demonstrating that Na^+^ can bind and activate the pump. Although the simulations predict E343^+^_E795^+^_E936^+^ to be K^+^ selective, the ATPase measurements indicate that E343Q_E795Q_E936Q is Na^+^ sensitive.

### Na^+^-sensitive protonation states $\left({\Delta \Delta G}_{binding}^{\left(K^{+}\to {Na}^{+}\right)}<2\;kcal/mol\right)$

**Figure 3:**
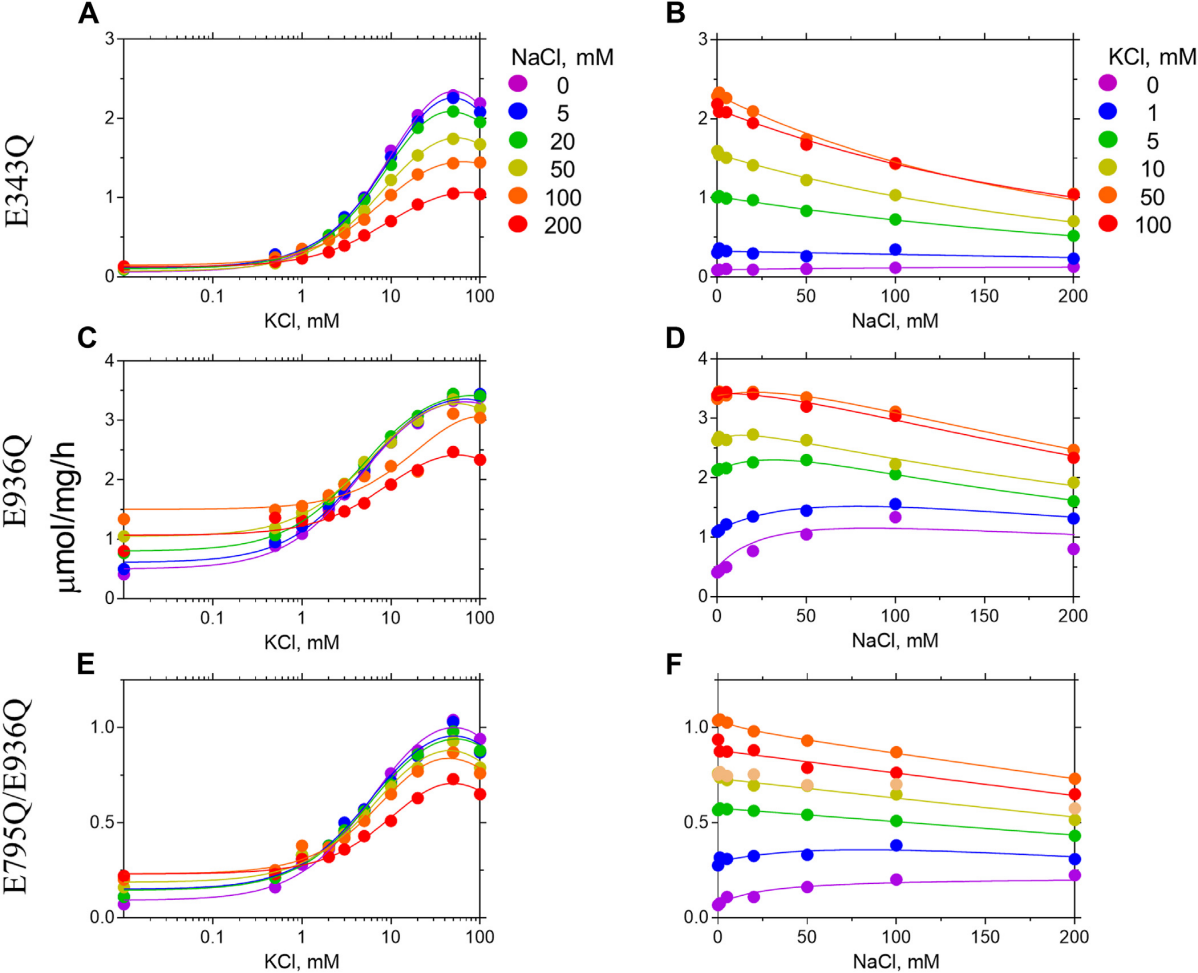
**ATPase activities of the indicated mutants.** (*A-F*) are shown in Figure 2.

E343 and E795 are not simultaneously protonated in any of the Na^+^-sensitive protonation states. For example, both E343 and E795 are deprotonated in E936^+^ (${\Delta \Delta G}_{binding}^{\left(K^{+}\to {Na}^{+}\right)}=0.71\;kcal/mol)$, E343 remains deprotonated in E795^+^_E936^+^ (${\Delta \Delta G}_{binding}^{\left(K^{+}\to {Na}^{+}\right)}=-1.5\;kcal/mol$), and E795 is deprotonated in E343^+^_E820^+^_E936^+^ (${\Delta \Delta G}_{binding}^{\left(K^{+}\to {Na}^{+}\right)}=0.99\;kcal/mol$). Although the V_max_ of E936Q ATPase activity (corresponding to E936^+^ in simulation) decreases with increasing [Na^+^] (Fig. 3*C*), the ATPase activity increases with increasing [Na^+^] in the low K^+^ concentration (∼10 mM KCl) regimen (Fig. 3*D*). If Na^+^ had acted as a substitute for H^+^ instead of K^+^ in H^+^,K^+^-ATPase, then one would observe an increase in *V*_max_ as seen in the sodium pump–like nongastric proton pump (16). Na^+^-dependent ATPase activation is observed only at low KCl concentration, and the extent of Na^+^ activation decreases with increasing K^+^ concentration. For example, in Figure 3*D*, maximum Na^+^-dependent ATPase activity at 0 mM KCl (violet circles) is approximately 1.3 μmol/mg/h. Since the basal H^+^-ATPase activity is approximately 0.5 μmol/mg/h, the extent of Na^+^ activation is 1.3 to 0.5 = 0.8 μmol/mg/h. This activation is further reduced to 0.5 (1.5–1.0) μmol/mg/h in the presence of 1 mM KCl (blue circles). These data suggest that Na^+^ binds instead of K^+^ to induce dephosphorylation of E2P, and therefore the rate constant is lower than that induced by the innate substrate K^+^. Therefore, E936Q is Na^+^ sensitive unlike the WT and the other single mutants, in agreement with the free energy calculations. Simulations predict E343^+^ to be Na^+^ sensitive, but the ATPase activity of E343Q (corresponding to E343^+^ in simulations) is similar to that of the wildtype (Fig. 3, *A* and *B*). We remind the reader that the ionization states of residues other than the mutated residue (E343 in the case of the E343Q mutant) cannot be resolved in experiments. Therefore, the E343Q state can correspond to E343Q_E795^+^, because the pKa of E795 is 13.15. In this way, the apparent disagreement between experiments and simulations can be reconciled for E343Q (E343^+^ in simulations). Like E936Q, E795Q_E936Q is also Na^+^ sensitive (Fig. 3, *E* and *F*), although the activity of E795Q_E936Q is lower than that of the WT. Altogether from these data we conclude that Na^+^ sensitivity is linked to the protonation of E936. In simulations, however, E343^+^_E795^+^_E936^+^ and E343^+^_E795^+^_E820^+^_E936^+^ are exceptions to this trend.

**Figure 4:**
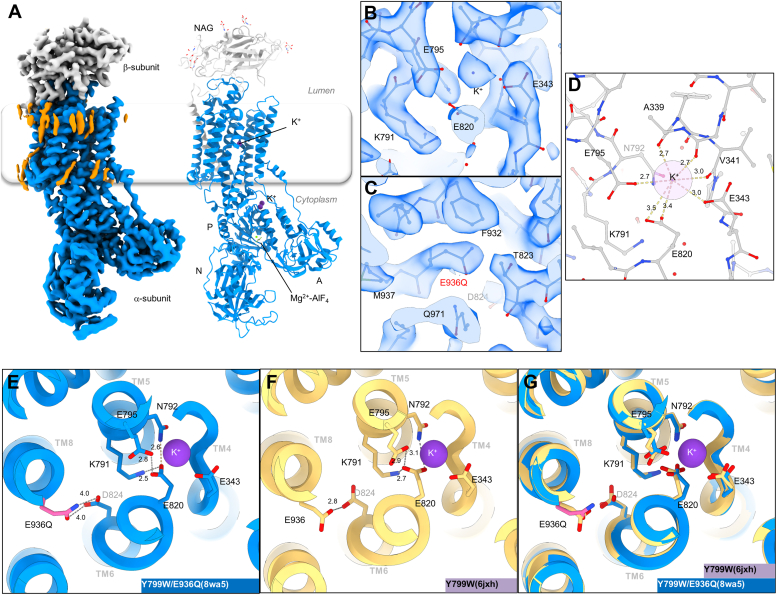
**Cryo-EM structure of the Y799W/E936Q mutant of gastric H**^**+**^**,K**^**+**^**-ATPase.***A*, EM potential map (colored surface) and cartoon model of the Y799W/E936Q mutant in the presence of 1 mM AlF_4_^−^ and 200 mM KCl to induce the (K^+^)E2-P_i_ state. The α-subunit is shown in *blue*, the β-subunit is shown in *gray*, and lipid or detergent-like densities are shown in *orange*. Bound ions are shown as *spheres* (*purple*, K^+^; *green*, Mg^2+^). AlF_4_^−^, six *N*-linked glycans are shown as *sticks*. *B* and *C*, extracellular view of the cation-binding site (*B*) and region where E936Q is located (*C*), showing the model (*sticks*) is well fit to the density maps (*transparent blue surface*). *D*, coordination geometry of K^+^ at the cation-binding site. *Dotted lines* connect between K^+^ and surrounding oxygen atoms located within 4.0 Å. Distances are also indicated in the figure. *E*–*G*, cation-binding sites in the Y799W/E936Q mutant (*E*) or Y799W (*F*, 6jxh) and their superimposition (*G*), viewed from the luminal side. *Dotted lines* indicate potential hydrogen bonds (numbers indicate its distance) located within 3.5 Å. However, in the Y799W/E936Q mutant, D824 and E936Q are too far away from each other to form a strong hydrogen bond.

The E936Q mutant exhibits both Na^+^ activation (Fig. 3*D*) and reduced apparent affinity for K^+^ (Fig. 3*C*). Since the E936 residue is located more than 11 Å away from the K^+^-binding site, it is unlikely that this residue is directly involved in the cation coordination. This suggests allosteric effect of E936Q mutation at the cation-binding site. In order to address its molecular basis, we performed cryo-EM structural analysis of the E936Q mutant. Mutation of E936Q is introduced on the background of the previously reported Y799W mutant, which induces luminal gate closure (14). A grid was prepared in the presence of phosphate analogue AlF_4_ and K^+^, and a 2.5 Å cryo-EM map stabilized in the K^+^-occluded E2-P_i_ state (Figs. 4*A* and S2) was obtained. Consistent with the local resolution plot (Fig. S2), the bound K^+^, coordinating amino acid residues in the cation-binding site (Fig. 4*B*), and the E936Q mutation itself (Fig. 4*C*) are well resolved, indicating that the model is highly plausible. The overall structure of the E936Q/Y799W mutant is almost identical to that of the crystal structure of Y799W (6jxh) with an overall RMSD of 1.11 Å. Majority of the differences arise from the relative orientations of the cytoplasmic domains in the α-subunit and the ecto domain of the β-subunit, possibly due to the absence of crystal packing in the cryo-EM analysis (Fig. S2*D*). Indeed, when the two structures were aligned according to their TM helices, the RMSD value of the respective region was 0.38 Å, indicating that the structure of the cation-binding site in the transmembrane region is very similar to each other in the two mutants. In the Y799W crystal structure, E936 located at the TM8 makes a hydrogen bond with D824 in TM6 (Fig. 4*F*, 2.8 Å), while the E936Q side chain is slightly further away from D824 (4.0 Å) to form a strong hydrogen bond in the Y799W/E936Q mutant structure (Fig. 4*E*). Despite this difference, however, K^+^ coordination (Fig. 4*D*) at the cation-binding site is not very different (Fig. 4*G*) because the hydrogen bond between D824 and E936 is further away from the binding site to have any effect on the cation coordination. Nevertheless, the valence calculated from the distance between K^+^ and the oxygen atoms participating in K^+^ coordination is 0.85 for the Y799W/E936Q mutant, which is lower than the value of 1.05 obtained for the WT, suggesting that K^+^ coordination in the E936Q mutant is not as compact as in the case of WT (Table S2, see distance between K^+^ and indicated oxygen atoms). A speculative explanation is that the change in the hydrogen bond between E936 and D824 alters the position of TM5 where D824 in present, which subsequently affects the rotamer conformation or position of the E820 side chain (Fig. 4*G*), which is also located in TM5 and involved in the K^+^ coordination. We, therefore, conclude that the observed loose coordination of the K^+^ in the Y799W/E936Q mutant structure may contribute to the reduced K^+^ affinity and Na^+^ selectivity of the E936Q mutant based on the static cryo-EM structures.

E820Q has too low an activity for interpreting ion selectivity. All variants containing the E820Q mutation (E795Q_E820Q, E343Q_E820Q, E820Q_E936Q, E343Q_E820Q_E936Q, and E795Q_E820Q_E936Q) have low to no activity (Fig. S3). We speculate that the E820Q mutation alters the pump-binding site rendering it incapable of ion binding or that the ion coordination after binding cannot initiate luminal gate closure or further dephosphorylation of the protein. It is also worth considering the possibility that the protonation or deprotonation of E820 is a necessary step for the completion of the pump cycle. Alternatively, the mutation may impede the formation of the salt bridge with K791, known to be critical for in the transport cycle (17, 18). As noted earlier, all variants containing the D824N mutation have negligible ATPase activity (Fig. S3). We have previously speculated that a D824-K791 salt bridge may stabilize the E1 state of the pump (19). The D824N mutation can lead to destabilization of the salt bridge and complete loss of function in the ATPase assays. Although the mutants E820Q and D824N exhibit low activity, they displayed similar expression levels and retention times as those of WT in a fluorescence detection size-exclusion chromatography with Superose6 column suggesting that the mutations did not cause any denaturation of the protein.

Of the two protonation states that are weakly K^+^ selective ($2\frac{kcal}{mol}<\;{\Delta \Delta G}_{binding}^{\left(K^{+}\to {Na}^{+}\right)}<4kcal/mol)$, E343 and E795 are simultaneously protonated only in E343^+^_E795^+^_E820^+^_E936^+^. E795^+^ is mildly K^+^ selective, although both E795 and E343 are not simultaneously protonated.

### Comparison of effect of protonation and charge-neutralizing mutation

**Figure 5:**
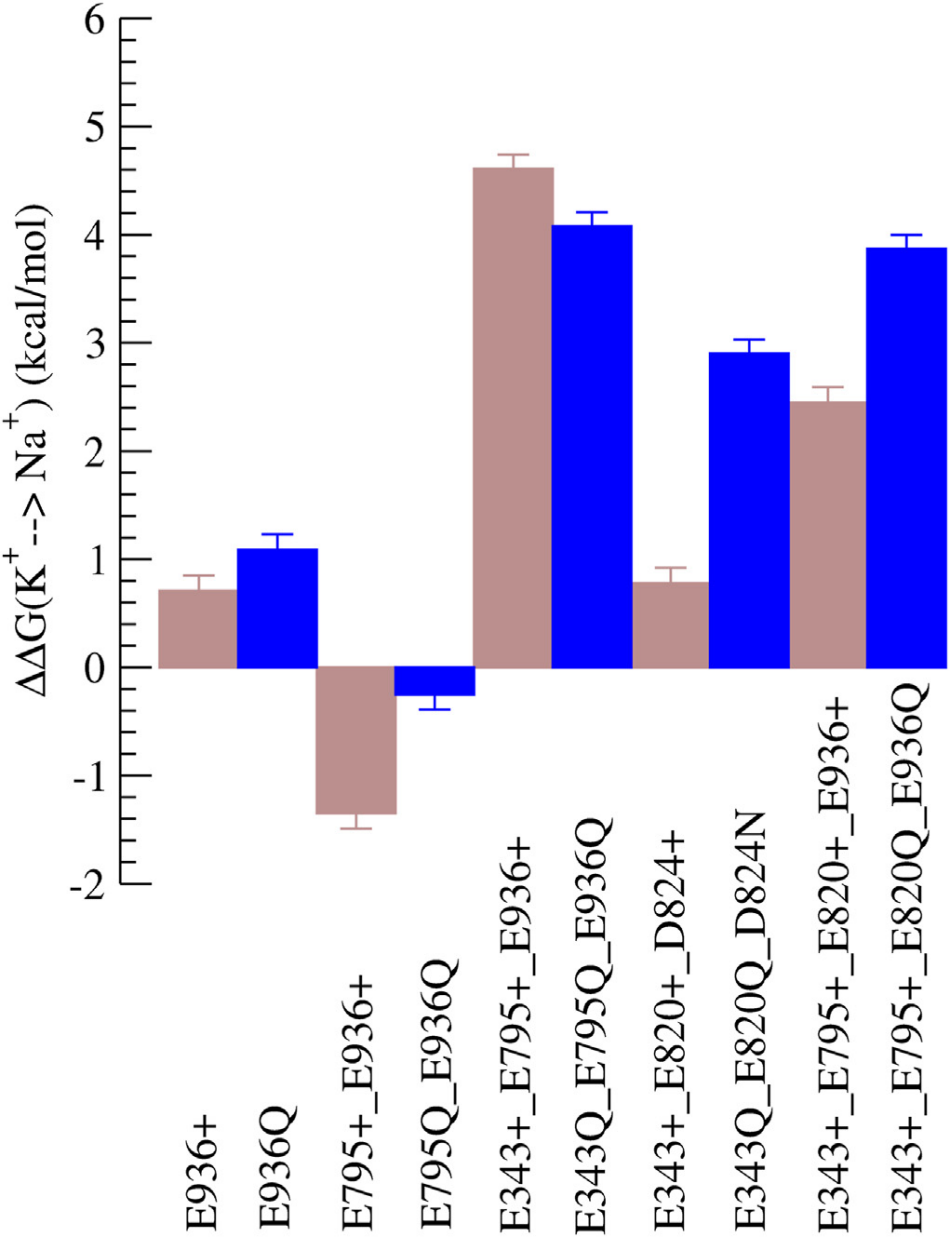
**Comparison of different protonation states and their corresponding charge-neutralizing mutations on the values of the relative free energy of ion binding**.

In the FEP calculations, we protonated the different binding-site residues, whereas in the ATP assay measurements, the Glu → Gln and Asp → Asn mutations mimic protonation. Protonation and mutation can have different ion coordination in the binding site, because a protonated acidic residue (Glu or Asp) is marginally chemically different from its neutral counterpart (Gln or Asn). The simulations predict E343^+^_E795^+^_E936^+^ to be K^+^ selective, while the ATPase measurements indicate that E343Q_E795Q_E936Q is Na^+^ sensitive. To attempt to resolve this disagreement, we compared the effect of protonation and charge-neutralizing mutations on ${\Delta \Delta G}_{binding}^{\left(K^{+}\to {Na}^{+}\right)}$ (Fig. 5). Replacing protonation of acidic residues by the corresponding charge neutralizing mutation does not alter the ${\Delta \Delta G}_{binding}^{\left(K^{+}\to {Na}^{+}\right)}$ values significantly. For example, ${\Delta \Delta G}_{binding}^{\left(K^{+}\to {Na}^{+}\right)}=4.26\;kcal/mol$ for E343^+^_E795^+^_E936^+^ and ${\Delta \Delta G}_{binding}^{\left(K^{+}\to {Na}^{+}\right)}=4.08\;kcal/mol$ for E343Q_E795Q_E936Q.

**Figure 6:**
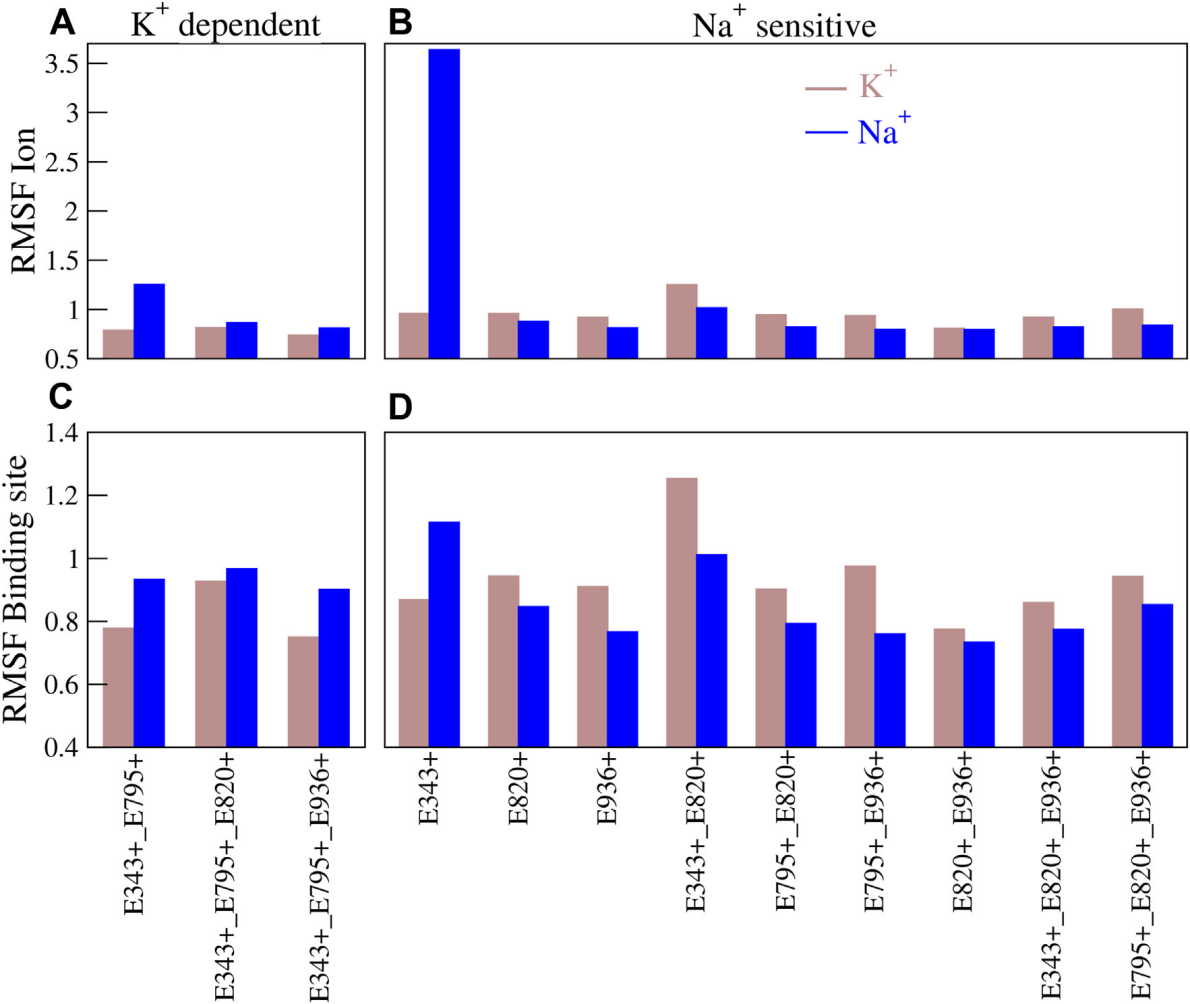
**Fluctuations of ions and binding site.***Top*: RMSF of the bound ions in (*A*) K^+^-selective protonation states and (*B*) Na^+^-sensitive protonation states. *Bottom*: RMSF of the heavy atoms of the binding-site residues in (*C*) K^+^-selective protonation states and (*D*) Na^+^-sensitive protonation states.

What is the molecular basis of the impact of the protonation state on the relative binding affinities of the ions and consequently the ion selectivity of the pump? We calculated the root mean square fluctuation of the ion and the heavy atoms of the residues at the binding site (Fig. 6). Na^+^ exhibits higher fluctuation than K^+^ in the K^+^-selective protonation states, while K^+^ fluctuates more than Na^+^ in the Na^+^-sensitive protonation states. Similarly, the fluctuation of the binding-site residues is higher when Na^+^ is bound in K^+^-selective protonation states or when K^+^ is bound in Na^+^-sensitive protonation states. Na^+^ bound in E343^+^ exhibits a large fluctuation because the ion moves from the initial binding site to site III during the simulation. Overall, binding of the correct ion leads to a more rigid binding site and corresponds to a higher free energy of binding.

The ATPase activity measurements indicate that E936Q and E795Q_E936Q exhibit Na^+^-sensitive ATPase activity. The relative free energy of binding obtained for the corresponding systems in the simulation (E936^+^ and E795^+^_E936^+^) agrees well with these findings. However, in the simulation, E343^+^_E795^+^_E936^+^ shows a preference to bind K^+^, whereas E343Q_E795Q_E936Q shows Na^+^-dependent pump activity. There can be several explanations for the apparent discrepancy between experiments and simulations. The structural and steric differences in the binding site caused by mutation instead of protonation are ruled out because we find no difference in relative ion affinities when comparing mutations with protonation (Fig. 5). Although the K^+^ binding affinity is higher than Na^+^ binding affinity in E343Q_E795Q_E936Q or E343^+^_E795^+^_E936^+^, the binding affinity of Na^+^ is still high enough for Na^+^ to induce ATPase activity in the ATPase assays. Another possibility is that the protonation states of the other acidic residues (*e.g.*, E820) are different in the protein in the activity measurements, thus making it difficult to directly compare E343Q_E795Q_E936Q with E343^+^_E795^+^_E936^+^. Although the same argument pertains to all other protonation states, it is possible that only the triple mutant leads to significant changes in the ionization states of the other amino acids in the binding site. It should also be noted that the free energy calculations predict differences in ion-binding affinity but cannot predict if the protein will continue to the next steps in the conformational cycle, which leads to ATP hydrolysis. Roux and coworkers (12) demonstrated in the Na^+^/K^+^ pump that, when the incorrect ion binds at the binding site, the free energy for the gate occlusion step increases as a self-correcting mechanism of ion binding. Therefore, a high K^+^ binding affinity in E343^+^_E795^+^_E936^+^ may not lead to gate closure and would result in low ATPase activity, as measured in the experiments. PROPKA predicts a pKa of 10.73, but our data clearly show that protonation of E936 is likely to lead to Na^+^ sensitivity. pKa estimates from single structures must therefore be interpreted carefully.

In the ATP activity measurements, we observe that Na^+^ has an inhibitory effect on the WT pump. The pump is able to resist an Na^+^ concentration of 100 mM in the stomach. If this concentration temporarily increases during a Na^+^-rich diet, then theoretically Na^+^ can inhibit acidification by the pump. However, doubling the concentration of Na^+^ in a 1.5-L stomach will necessitate consumption of ∼3.5 g of salt in a single meal, while WHO recommends a maximum intake of less than 2 g of Na^+^ per day. Even a bag of potato chips contains ∼120 mg Na^+^, which will only increase the Na^+^ concentration in the lumen by 3.5%. Therefore, dietary Na^+^ is unlikely to have an immediate impact on stomach lumen acidification by the HKA. However, extremely high sodium consumption may have an inhibitory effect on the proton pump and requires further investigation.

There are two common themes that emerge from the calculations and analytical measurements. First, as predicted in the past (14, 20), both E343 and E795 need to be protonated simultaneously to induce K^+^ selectivity. Secondly, E936 is protonated in most of the protonation states that are Na^+^ sensitive in simulations. The exceptions to this rule in simulations are E343^+^_E795^+^_E936^+^ and E343^+^_E795^+^_^+^E820^+^_E936^+^, and the former is predicted to be Na^+^ sensitive in ATPase activity measurements. Taken together, our data indicate that Na^+^ sensitivity arises from the protonation of E936, while protonation of E795 and E343 is required for K^+^ selectivity.

## Experimental procedures

### System preparation for simulations

The protein coordinates in the E2 occluded state was obtained from the PDB ID (6jxh). The detergents present in the PDB file were removed, and the MgF_4_^2−^ was replaced with a PO_4_^3−^ molecule. The glutamic acid and aspartic acid residues facing the lumen were protonated. The protein was then oriented using the OPM (21) server and subsequently inserted into a bilayer consisting of ∼500 1,2-dioleoyl-sn-glycero-3-phosphocholine lipid molecules using the CHARMM-GUI server (22, 23, 24). The system was solvated with TIP3P (25) water molecules, and a physiological ion concentration was maintained by adding 0.15 M NaCl. The resulting system was energy minimized using the steepest descent method. The system was then equilibrated in three short NVT and NPT simulations where the restraints on the heavy atoms of membrane and protein were gradually released. The complex was then simulated with a timestep of 2 fs for 100 ns without any restraints under NPT conditions. The temperature and pressure were maintained at 310 K and 1 bar using the Nose–Hoover (26, 27) thermostat and the Parinello–Rahman (28) barostat, respectively. Such a 100 ns simulation was performed for all protonation states considered in this work. The electrostatic interactions were treated using the particle-mesh Ewald (29) method with a short-range cutoff of 1.2 nm, and the van der Waals interactions were smoothly switched off between 1.0 nm and 1.2 nm using a force-switch function. All the simulations were performed in GROMACS (30) v. 2020.4 employing the CHARMM36 (31, 32, 33) force field.

### Free energy perturbation simulations

**Figure 7:**
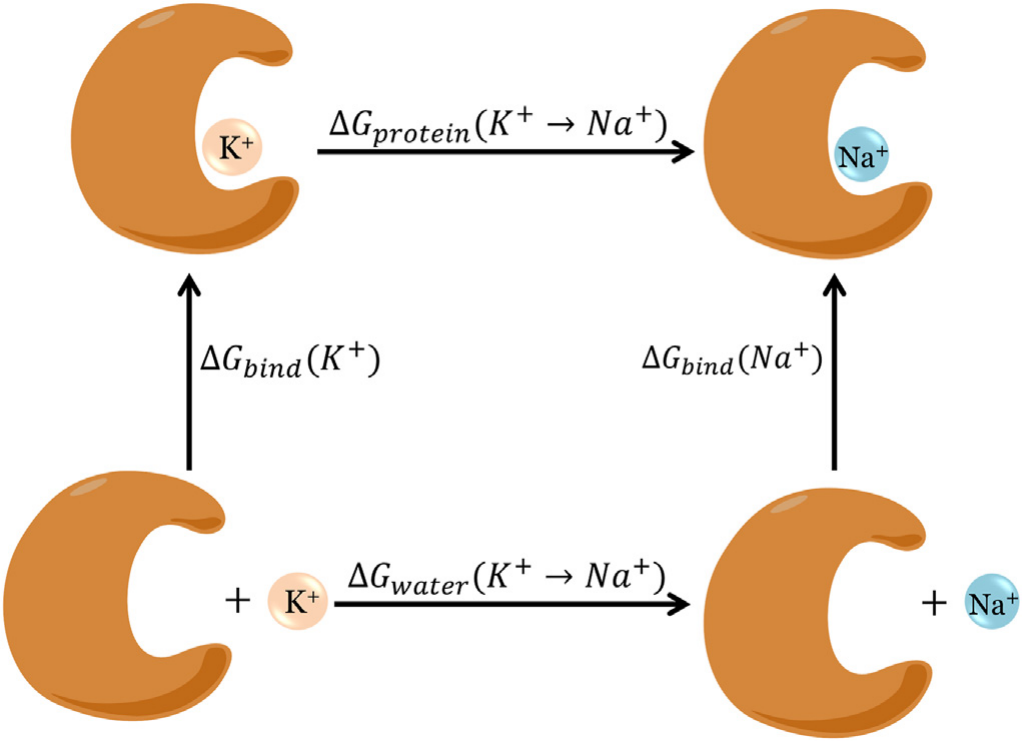
**The thermodynamic cycle used for the transformation of the cation to obtain the difference in the binding free energy.** The free energies for the horizontal reactions are evaluated to measure the free energy difference.

To calculate the differences in the binding affinity of K^+^ and Na^+^ to the protein, we employed the FEP method to calculate the energy changes associated with the transformation of K^+^ → Na^+^ bound at the ion-binding site. The thermodynamic cycle is shown in Figure 7. The relative binding of free energy of the ions is given as ${\Delta \Delta G}_{binding}^{\left(K^{+}\to {Na}^{+}\right)}=\Delta G_{bind}\left({Na}^{+}-K^{+}\right)=\Delta G_{protein}\left(K^{+}\to {Na}^{+}\right)-\Delta G_{water}\left(K^{+}\to {Na}^{+}\right)$. A positive value of ${\Delta \Delta G}_{binding}^{\left(K^{+}\to {Na}^{+}\right)}$ indicates that K^+^ binds more favorably than Na^+^ and that the mutant is K^+^ selective. The final coordinates of the systems obtained after the classical 100 ns simulations were used as the initial coordinates for the FEP simulations. The transformation was performed in 26 equally spaced steps where the nonbonded parameters of K^+^ were interpolated to that of Na^+^ using the single-topology method. Each of the 26 windows was energy minimized and subsequently simulated for 15 ns. The first 5 ns were discarded for the final free energy difference calculation. The Alchemical analysis tool (34) was then used to calculate the free energy difference between the two end states employing the multistate Bennett acceptance ratio (35) method. In water, the same transformation yielded a value of −18.5 kcal/mol, which is in good agreement with the previously reported simulation and experimental value (−17.5 kcal/mol) (13, 36).

### Protein expression and purification for the structural analysis

To obtain the cryo-EM structure of the Glu936Gln (E936Q) mutant, procedures for protein expression were followed as those reported (19, 37). Briefly, the first 48 amino acids from the N terminus of the pig gastric H^+^,K^+^-ATPase mutant (Tyr799Trp—this mutation stabilizes the K^+^-occluded E2-AlF form (14) and Glu936Gln) were deleted and replaced with a Flag epitope tag (DYKDDDDK), a hexa-histidine tag, and the enhanced green fluorescence protein (EGFP). A tobacco etch virus (TEV) protease recognition sequence was also added so that the final construct read as Met-Flag(DYKDDDDK)-(His)_6_-EGFP-TEV-Δ48-Y799W/E936Q/H^+^,K^+^-ATPase. This was subcloned into a handmade vector. The wildtype pig gastric H^+^,K^+^-ATPase β-subunit was also cloned. The αβ-complex of gastric H^+^,K^+^-ATPase was expressed in the plasma membrane using baculovirus-mediated transduction of mammalian HEK293S GnT1**^−^** cells (38) purchased from ATCC.

For cryo-EM analysis, cells were directly solubilized with 1% lauryl maltose neopentyl glycol (39) in the presence of 40 mM Mes/Tris (pH 6.5), 10% glycerol, 5 mM dithiothreitol, 1 mM MgCl_2_, 1 mM AlCl_3_, 4 mM NaF, 200 mM KCl, and protease inhibitor cocktail (Roche) on ice for 20 min. After removing insoluble material by ultracentrifugation, the supernatant was mixed with anti-Flag M2 resin at 4 °C for 2 h, which was followed by washing with buffer containing 40 mM Mes/Tris (pH 6.5), 1% glycerol, 1 mM MgCl_2_, 1 mM AlCl_3_, 4 mM NaF, 200 mM KCl, and 0.06% glyco-diosgenin (40). Elution fractions from Flag resin with 0.2 mg/ml Flag peptide were incubated with TEV protease and endoglycosidase at 4 °C overnight. Digested peptide fragments containing EGFP and endoglycosidase were removed by passing the fractions through Ni-NTA resin (Qiagen). Flow-through fractions were concentrated and subjected to size-exclusion column chromatography using a Superose6 Increase column (Cytiva) equilibrated in buffer comprising 20 mM Mes/Tris (pH 6.5), 1 mM MgCl_2_, 1 mM AlCl_3_, 4 mM NaF, 200 mM KCl, and 0.06% glyco-diosgenin. Peak fractions were collected and concentrated to 8 mg/ml.

### Cryo-EM analysis

Preparation of samples and cryo-EM grids were done according to a previous report (16). The purified protein samples (at 8 mg/ml) were applied to freshly glow-discharged Quantifoil holey carbon grids (R1.2/1.3, Cu/Rh, 300 mesh), using a Vitrobot Mark IV (Thermo Fisher) at 4 °C with a blotting time of 4 s under 99% humidity and then plunge-frozen in liquid ethane. The prepared grids were transferred to a Titan Krios G3i microscope (Thermo Fisher Scientific), operated at 300 kV, and equipped with a Quantum-LS Energy Filter (GIF) and a Gatan K3 Summit direct electron detector in the electron counting mode. Imaging was performed at a nominal magnification of 105,000×, corresponding to a calibrated pixel size of 0.83 Å/pix (The University of Tokyo). Each movie was recorded in correlated-double sampling mode for 3.2 s and subdivided into 54 frames. The electron flux was set to 14 e^−^/pix/s at the detector, resulting in an accumulated exposure of 64 e^−^/Å^2^ at the specimen. The data were automatically acquired by the image shift method using SerialEM software (41), with a defocus range of −0.8 to −1.8 μm. The dose-fractionated movies were subjected to beam-induced motion correction, using RELION 3.1 (42), and the contrast transfer function (CTF) parameters were estimated using patch CTF estimation in cryoSPARC (v4, Structura Biotechnology) (43).

Particles were initially picked by blob picker using cryoSPARC (v4) and extracted with down-sampling to a pixel size of 3.32 Å/pix. These particles were subjected to several rounds of 2D classifications. Good-looking classes were then subjected to *ab initio* reconstruction in three models and refined by nonuniform refinement (43). The particles from the best class were then re-extracted to the full pixel size and subjected to nonuniform refinement with per-particle defocus refinement, beam-tilt refinement in cryoSPARC (v4). The particle stack was then transferred to RELION 3.1 and subjected to Bayesian polishing (44). Polished particles were reimported to cryoSPARC (v4) and subjected to nonuniform refinement. The resolution of the analyzed map was defined according to the FSC = 0.143 criterion (45) (Fig. S2 and Table S1). The local resolution and angular distributions for each structure were estimated by cryoSPARC (v4). All the models were manually built in Coot (46) using a crystal structure of K^+^-occluded E2-MgF form of gastric H^+^,K^+^-ATPase (6jxh) as a starting template (14). Phenix (version 20) (47) was used for refinement. The model (PDB ID: 8WA5, https://doi.org/10.2210/pdb8wa5/pdb) and EM density map (EMDB code: EMD-37391) have been deposited in protein data bank and EM data base, respectively.

### ATP assays for measuring activity

To interpret the simulation results, we performed ATP assay experiments to calculate the ATPase activity of the protein. Since it is nearly impossible to identify the protonation state of the acidic residues in the experiment, we implement charge-neutralizing mutations (E → Q and D → N) to mimic protonation of the acidic residues. The wildtype or mutant α-subunit was coexpressed with the wildtype β-subunit using the BacMam as described above, and broken membrane fractions were collected (48). The ATPase activity was measured as described (48). Briefly, permeabilized membrane fractions were suspended in buffer comprising 40 mM Pipes/Tris (pH 7.0), 2 mM MgCl_2_, 2 mM ATP (Tris salt), in the presence of indicated concentrations of NaCl and KCl, in 96-well tubes. We added 10 μM vonoprazan (specific inhibitor for H^+^,K^+^-ATPase) for 0 mM KCl and different concentrations of NaCl conditions as blanks. Reactions were initiated by incubating the fractions at 37 °C using a thermal cycler and maintained for 1 to 3 h depending on their activity. Reactions were terminated, and the amount of released inorganic phosphate was determined colorimetrically (49) using a microplate reader (TECAN).

## Data availability

Atomic coordinates and EM density maps are available at PDB and EMDB under the accession code 8WA5, EMDB-37391: cryo-EM structure of the gastric proton pump Y799W/E936Q mutant (validation report for the structure is available).

## Supporting information

This article contains supporting information.

## Conflict of interest

The authors declare that they have no conflicts of interest with the contents of this article.
