# Supplementary material for: Specific protonation of acidic residues confers K+ selectivity to the gastric proton pump
Source: J Biol Chem. 2023 Dec 10;300(1):105542. doi: 10.1016/j.jbc.2023.105542 (PMC10825007; doi:10.1016/j.jbc.2023.105542)
Supplement: Supporting Figures S1–S3 and Table S1–S2 [file mmc1.docx]

**Table S1: Cryo-EM data collection, refinement, and validation statistics**

|  | Gastric proton pump Y799W/E936Q mutant  (K^+^)E2-AlF form  (EMDB-37391)  (PDB 8WA5) |
| --- | --- |
| **Data collection and processing** |  |
| Magnification | 105,000 |
| Voltage (kV) | 300 |
| Electron exposure (e^–^/Å^2^) | 64 |
| Defocus range (μm) | 0.8-1.6 |
| Pixel size (Å) | 0.83 |
| Symmetry imposed | *C1* |
| Initial particle images (no.) | 1,025,327 |
| Final particle images (no.) | 302,608 |
| Map resolution (Å)  FSC threshold | 2.51  0.143 |
|  |  |
| **Refinement** |  |
| Initial model used (PDB) | 6jxh |
| Model resolution (Å)  FSC threshold | 2.6  0.5 |
| Map sharpening *B* factor (Å^2^) | -88.3 |
| Model composition  Non-hydrogen atoms  Protein residues  Waters  Ligands | 9,929  1,249  6  3K^+^, Mg^2+^, ALF, PCW, CLR, 5NAG |
| *B* factors (Å^2^)  Protein  Ligand  Waters | 54.10  53.50  29.52 |
| R.m.s. deviations  Bond lengths (Å)  Bond angles (°) | 0.003  0.558 |
| Validation  MolProbity score  Clashscore  Poor rotamers (%) | 1.34  4.94  0.85 |
| Ramachandran plot  Favored (%)  Allowed (%)  Disallowed (%) | 97.59  2.41  0.0 |

**Table S2: Coordination geometry and partial valence at the K^+^-binding site. Only oxygen atoms within 4 Å of K+ are included for the valence calculation as in ref 14.**

|  | Y799W/E936Q | | Y799W* | |
| --- | --- | --- | --- | --- |
|  | Distance, Å | Partial valence | Distance, Å | Partial valence |
| V338 O | 3.01 | 0.08 | 2.90 | 0.11 |
| A339 O | 2.67 | 0.23 | 2.62 | 0.28 |
| V341 O | 2.70 | 0.21 | 2.56 | 0.34 |
| E343 Oε1 | 3.00 | 0.08 | 2.93 | 0.10 |
| E343 Oε2 | 4.20 | - | 3.74 | 0.01 |
| E795 Oε1 | 2.72 | 0.20 | 2.77 | 0.17 |
| E820 Oε1 | 3.36 | 0.03 | 3.32 | 0.03 |
| E820 Oε2 | 3.47 | 0.02 | 3.90 | 0.01 |
| Total valence | | 0.85 |  | 1.05 |

*from ref 14


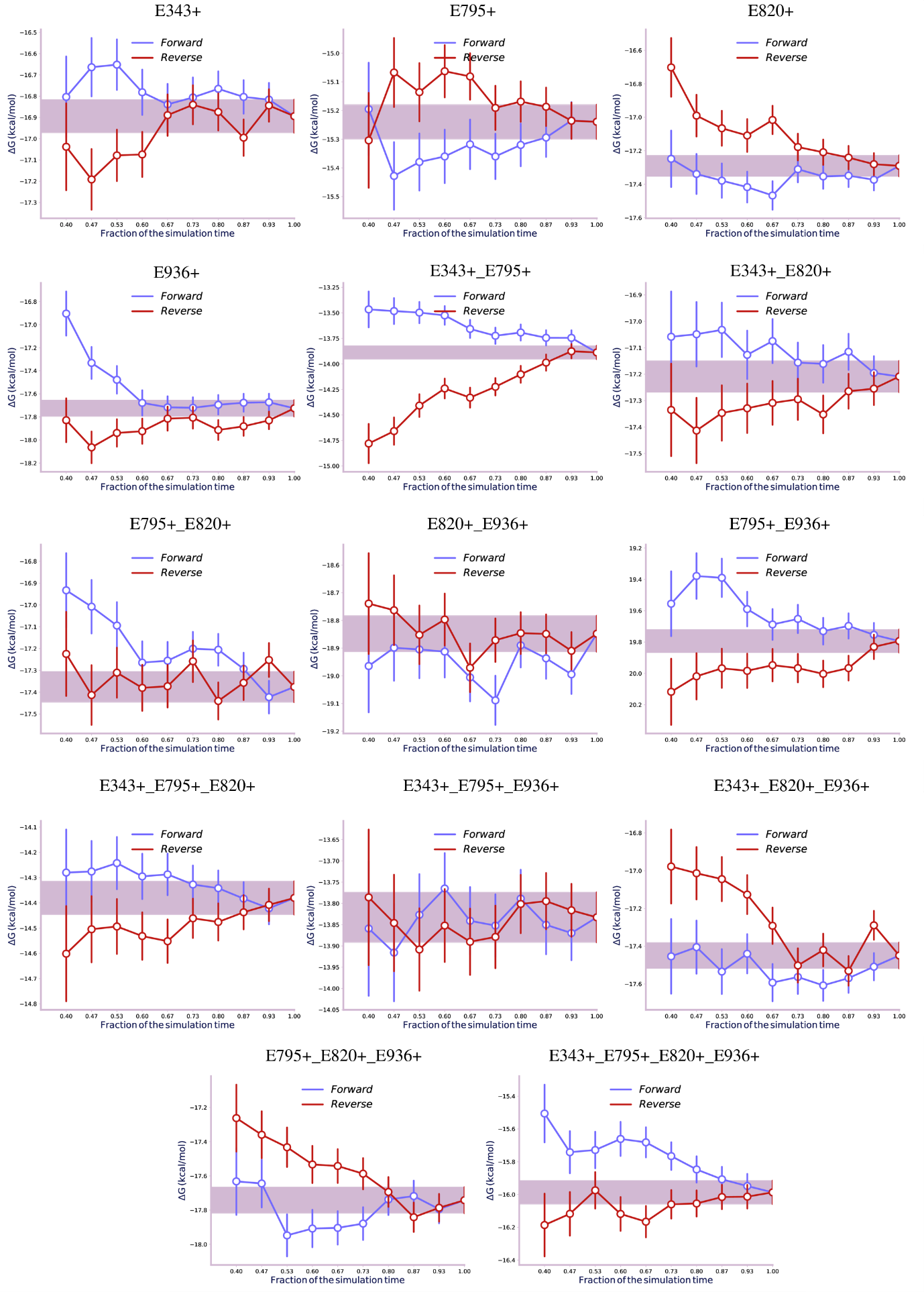


Figure S1: Estimated free energy change using a fraction of simulation for each protonation state. There are 26 lambda states and each lambda state is simulated for 15 ns. The first 5 ns of each lambda state is discarded as equilibration data during free energy calculation.


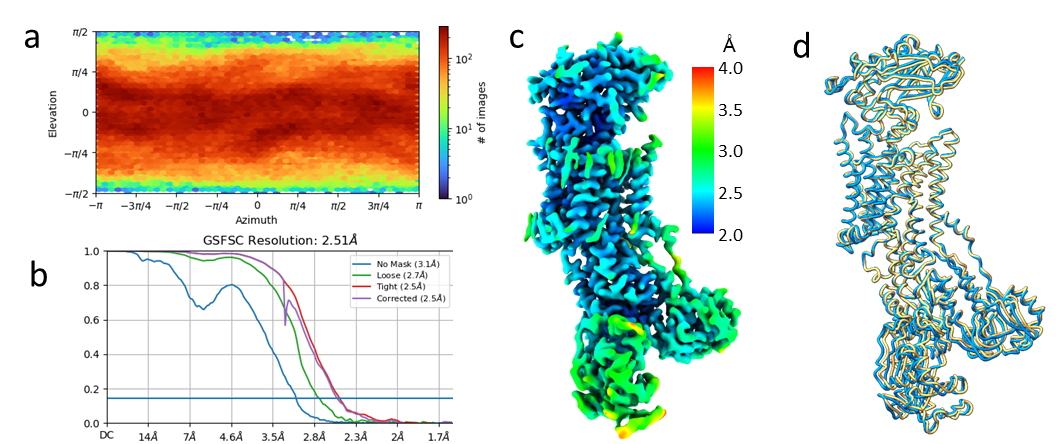


Figure S2: Cryo-EM analysis of HKA Y799W/E936Q mutant. (a) Angular distribution plot of particles included in the final 3D reconstruction. The number of views at each angular orientation is represented by the color (blue to red). (b) FSC plot used for resolution estimation. Blue line indicates FSC value of 0.143. (c) An unsharpened map colored by local resolution as calculated by cryoSPARC (scale is indicated in the figure). (d) Comparison of the molecular conformations between HKA Y799W (wheat) and Y799W/E936Q (blue).


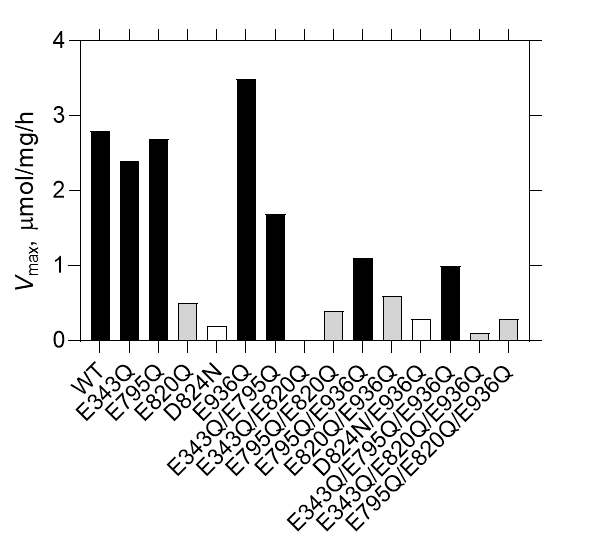


Figure S3: The maximum ATPase activities of indicated mutants were plotted from their K^+^-dependent ATPase measurement (Figure 2 and Figure 3). Mutants containing E820Q (grey columns) or D824N (white columns), that exhibit low activity, were plotted in different colours.
